# Dietary protein safety and resistance exercise: what do we really know?

**DOI:** 10.1186/1550-2783-6-3

**Published:** 2009-01-12

**Authors:** Lonnie M Lowery, Lorena Devia

**Affiliations:** 1 Nutrition Assessment Laboratory, Nutrition Center, 210 Schrank Hall South, University of Akron, Akron, Ohio, 44325-6103, USA; 2 Nutrition Exercise and Wellness, Inc. P.O. Box 228 Cuyahoga Falls, Ohio, 44222, USA

## Abstract

Resistance trainers continue to receive mixed messages about the safety of purposely seeking ample dietary protein in their quest for stimulating protein synthesis, improving performance, or maintaining health. Despite protein's lay popularity and the routinely high intakes exhibited by strength athletes, liberal and purposeful protein consumption is often maligned by "experts". University textbooks, instructors, and various forms of literature from personal training groups and athletic organizations continue to use dissuasive language surrounding dietary protein. Due to the widely known health benefits of dietary protein and a growing body of evidence on its safety profile, this is unfortunate. In response, researchers have critiqued unfounded educational messages. As a recent summarizing example, the *International Society of Sports Nutrition (ISSN) Position Stand: Protein and Exercise *reviewed general literature on renal and bone health. The concluding remark that "Concerns that protein intake within this range [1.4 – 2.0 g/kg body weight per day] is unhealthy are unfounded in healthy, exercising individuals." was based largely upon data from non-athletes due to "a lack of scientific evidence". Future studies were deemed necessary. This assessment is not unique in the scientific literature. Investigators continue to cite controversy, debate, and the lack of direct evidence that allows it. This review discusses the few existing safety studies done specific to athletes and calls for protein research specific to resistance trainers. Population-specific, long term data will be necessary for effective education in dietetics textbooks and from sports governing bodies.

## Introduction

There appears to be an element of disconnectedness between scientific evidence and health messages offered to students and athletes. Statements of concern over the effects of ample dietary protein intakes appear in Table 1. Research on healthy populations, however, does not support such concerns. One summary of the literature on this topic, the *International Society of Sports Nutrition (ISSN) Position Stand: Protein and Exercise *[1] reviewed literature on renal and bone health, among other topics. Although balanced in its inclusion of both negative (no evidence of harm) and positive (extrapolated evidence of potential concern) studies, the position stand was largely without mention of athlete-specific data on safety topics. Examples of athlete-specific research, although rare, do exist and are included in this review. Three safety issues are commonly mentioned in popular media and nutrition and dietetic textbooks, while sports governing bodies may focus upon the risk of dietary supplements per se [2,3]. One issue is renal "stress", [2,4] a second issue is calcium loss and bone catabolism [2,5,6] and a third is an assumption that higher protein intakes are higher in saturated fat and lower in fiber [2]. Language surrounding these topics can be dissuasive and/or uncertain regarding purposeful consumption of protein for weight control or athletic reasons. (Table 1.) Although difficult to document due to its frequently verbal nature, this is a curious phenomenon considering the lack of evidence, particularly among strength athletes, who are widely known to pursue additional dietary protein for performance or body composition purposes [7].

**Table 1:** Protein-related statements in educational materials [2]

"Overconsumption of protein offers no benefits and may pose health risks. High protein diets have been implicated in several chronic diseases including heart disease, cancer, osteoporosis, obesity and kidney stones..."
"This section briefly describes the relationships between protein intake and bone loss. When protein intake is high calcium excretion rises."

"...people take these [protein] supplements for many different reasons, all of them unfounded... Like many other magic solutions to health problems, protein and amino acid supplements don't work these miracles [and] may be harmful."

"Normal, healthy people never need protein or amino acid supplements."

"Muscle work builds muscle; [protein] supplements do not..."

"Overconsumption of protein offers no benefits and may pose health risks."

"Excesses of protein offer no advantage; in fact, overconsumption of protein-rich foods may incur health problems as well."

"Athletes are not only pumping iron these days, they're also pumping protein supplements in hopes of building muscles..."

"If excess protein means excess calories, this adds weight as fat, not muscle"

"Purified protein supplements can contribute to calcium losses and therefore harm bone health."

"High protein diets have been implicated in the development of weak bones, kidney stones, cancer, heart disease and obesity."

"Diets very high in protein result in death after several weeks."

"Because information on the effects of high-protein intakes is limited, people are cautioned not to consume high levels of protein from foods or supplements."

"...intended to protect student-athlete well being..." and "A permissible supplement can contain no more than 30 percent of its calories from protein"; Other language in document: "protect", "warning", "potentially harmful", "risk", "concoction"

"Studies present conflicting data as to whether or not animal protein, as contrasted to plant protein, decreases bone density with an increased risk of osteoporosis and bone fractures."

"Taking large amounts of these supplements can lead to dehydration, loss of urinary calcium, weight gain, and kidney and liver stress."

"In fact, protein consumed in excess of what the body needs will be converted to fat."

"Also of concern is that excessive protein consumption can cause dehydration and place added stress on the kidneys and liver."

"There are a number of problems associated with excessive meat and protein consumption."

"The more protein you eat, the more calcium is excreted; this can compromise bone health."

"High protein diets also stress the kidneys, and may cause diarrhea and worsen dehydration."

"Excess protein in the diet is usually turned into fat, not muscle."

It is important to point out that quotes listed in Table 1 are not necessarily incorrect and may be followed or preceded by qualifying language. The statements do reflect an element of dissuasion (considering that overconsumption or excess of any nutrient is unhelpful or risky) and/or uncertainty (considering that studies present conflicting data or no information exists). Although the controversy is difficult to document, dissuasive viewpoints tend not to be seen as often in carbohydrate chapters.

## Protein intake and renal function in athletes

Protein amount and type may matter regarding renal function alterations in healthy persons, both acutely (single meals) and chronically [8]. These data appear inconsistent, however, and appear to depend on the population studied. Beyond study of chronically diseased persons or mixed groups of healthy persons, exercising populations should be studied specifically due to their known differences in renal function. Unfortunately, we simply do not know if these differences are helpful or harmful. Will biological differences among athletes lead to greater or less incidence in renal disease compared to the approximately 9% reported to develop among "normal healthy" persons [9]? Some differences among athletes suggest increased vulnerability to renal damage and others suggest protection against it. For example, it has been estimated that splanchic and renal tissues receive 50% of resting cardiac output (approximately 3 L per minute) whereas these tissues receive under 5% of cardiac output (less than 1 L) during maximal exercise due largely to sympathetic vasoconstriction [10]. Although exercisers adapt to some extent to this constriction, the relative ischemia may be detrimental to organ function [10]. Indeed, research documents incidences of extreme muscle damage and renal failure (rhabdomyolysis) in various sports, including bodybuilding [11]. Interestingly, protein intake may be a factor leading to associated creatine kinase elevations after resistance exercise [12].

On the other hand, less severe exercise and the resulting reduction in blood flow and filtration [13] may instead allow periods of "respite" for the kidneys. Periodic exercise sessions might reduce total renal work over time. Could this slow the normal age-related decline in glomerular filtration? It is not known. Data on exercise-related blood flow changes (sympathetic shunting) are largely animal based, leaving many unknowns among exercising humans.

The scientific community does know that exercising humans differ from non-exercisers in the amount of protein that can be found in their urine. Outside of the post-exercise period, both endurance trainers and resistance trainers exhibit lower microalbuminuria [14]. The reduction in this "damage marker" does appear beneficial. As with sympathetic shunting of blood flow, however, the full significance of this difference is not clear. At times, exercisers actually exhibit *increased *protein in their urine. The prevalence of proteinuria during and after exercise ranges from 18–100%, depending on exercise type and intensity – but not duration [15]. Thus, there are periods in an exerciser's day where there is more, not less of this renal "damage marker". It should be noted that, unlike the proteinuria seen after a protein-rich meal, post-exercise proteinuria is not considered damaging [16]. Still, the transient (~60 min half-time) post-exercise presence of protein in the urine [16] is clearly different from what a healthy non-exerciser would exhibit. Again, the populations differ.

Even exercisers are not uniform in their renal-vascular physiology. Resistance trainers, for example, not only exhibit intense muscular activity but also vascular changes which are different from endurance athletes [17]. Could large and repeated fluctuations in blood pressure, sympathetic activity, renal function, muscle microtrauma (creatine kinase concentrations), or even purposeful diet-induced hyper-insulinemia make this population different? Unfortunately, little to no research has compared renal function in groups of resistance trainers who have or have not sought ample dietary protein over a multi-year period. This absence of data is important because "education" provided to this population – which exhibits known differences in renal function – often involves concerned or dissuasive language [2]. Given the known benefits of dietary protein foods/supplementation on protein synthesis, athletic recovery and potentially weight control (thermic effect, satiety), among other potential benefits such as accompanying nutrients, elevated antioxidant capacity, immune enhancement and overtraining amelioration,[18] this "education" could be a mistake.

Two studies involving resistance trainers, specifically, are known to the authors of this review. These investigations will be examined in an effort to discern why their negative findings have not influenced educators' dissuasive language surrounding dietary protein. There will be a focus on population specificity and control variables as well as suggestions for future research.

The first relevant study on athletes was performed in Belgium by Poortmans and Dellalieux in 2000 [19]. This protocol detected no significant differences in renal function between higher and lower protein consumers. Despite being well controlled in most respects, there were a few issues of potential relevance to future study, particularly if it is to be longer-term. (Table 2.) Notably, the average-protein group was not from the same population as the higher-protein group. The average protein consumers were a collection of judoka, rowers and cyclists (skill and endurance-focused sports) while the group of higher protein consumers were bodybuilders (a strength and muscle mass-focused sport). Accordingly, the groups differed in 1.) Training stresses, 2.) Aerobic capacity, 3.) Body weight (presumably muscle mass) and 4.) Probably dietary practices. Over sufficient periods, could adaptations specific to heavy resistance training, such as vascular changes, affect study findings [17,20]? Should other, diverging physical or lifestyle issues be addressed in future, needed, longer-term investigations? The following delineates how these four issues might affect results. *Training stresses*: Mid-exercise differences such as blood flow variability, intra-abdominal pressures and extreme blood pressure changes occur among heavy lifting bodybuilders [21,22]. Although transient, this may matter because "central pressures are more closely related to the pathophysiology of end-organ damage [23]. Perhaps more importantly, arterial stiffness is exhibited by resistance trainers and this general condition has been associated with glomerular decline [17,20]. Would a study of sufficient duration detect an emergence of renal damage among bodybuilders first? And might this be a natural consequence of their sport, irrespective of protein intake? *Aerobic capacity*: Endurance athletes with high VO_2 _max can exhibit rhabdomyolysis just as bodybuilders do. Due to exercise-induced dehydration from long sessions or due to (arguably) possession of more muscle myoglobin, could endurance athletes be more likely to have myoglobin precipitate in their kidneys and harm renal function? Would a study of sufficient duration detect an emergence of renal damage among *these *athletes first? 3.) *Body weight/muscle mass*: The greater muscle mass of strength athletes may also affect findings over time. Lean body mass has been shown to influence serum creatinine concentrations and thus presumably renal "work" [19]. Indeed, lean body mass has been shown to influence renal function [24]. Muscle mass is also the primary recipient of blood glucose. Could intense exercise and repeated, whole-body eccentric muscle soreness (and thus transient insulin resistance) accelerate renal decline, due to associated hyperglycemia and hyperinsulinemia [25-27]? Perhaps this is another reason for population-specificity in future study designs. 4.) *Dietary practices*: In a population (bodybuilders) that already raises serum insulin with whey-carbohydrate drinks and large food intakes in general, any glycemic or insulinemic aberrations induced by muscle soreness may be particularly relevant. Hence, there are many physical activity and dietary parameters to consider [28]. In all, an appreciation of the differences among athletes may be of greater importance if longer-term and/or observational studies are undertaken.

**Table 2:** Methodological issues in existing protein-athlete investigations

**Higher-protein group**	**Lower-protein group(s)**	**Duration of higher-protein intake**	**Uncontrolled or unanalyzed variables**	**Nationality**	**Reference**
Large male bodybuilders (protein 169 ± 13 g/d)^1^	Smaller, male endurance and skill athletes (protein 99 ± 8 g/d)^1^	unspecified	Prior exercise, body composition,	Belgian	19

Large male bodybuilders (protein 142 ± 75 g/d)^2^	Smaller, mixed male and female bodybuilders, vegetarians, "normals" (protein 84 ± 35 g/d)^2^	As little as four months	Prior exercise^3^, body composition, non-protein nutrition info. (diet logs)	German	30

1. Relative protein intake 1.94 ± 0.13 g/kg daily (Higher group) vs. 1.35 ± 0.12 g/kg daily (Lower group) 2. Relative protein intake 1.65 ± 0.87 g/kg daily (Higher group) vs. 1.41 ± g/kg daily (Lower group) 3. Exercise not specified but catabolic events were controlled.

The second relevant study on athletes was performed in Germany by Brandle and colleagues [29]. The investigators found no correlation between albumin excretion rate (urinary albumin arguably being a damage variable) and gross protein intake (as assessed by nitrogen excretion rate). This investigation was also carefully done in many respects but left room for future research. (Table 2.) Again, the average-protein groups differed from the higher protein group, as opposed to being from the same population. The average protein consumers (comparison groups) were of different types: non-supplementing bodybuilders, vegetarians and normal healthy persons. These average-protein groups differed in weight, sex, serum creatinine, serum urea, and in two instances physical activity, from the higher-protein group. Perhaps most importantly, the subjects had been on their present diet for as little as four months. Although longer term than previous 1–4 week studies and long enough to see hormonal or perhaps structural changes in the kidney, it is not known whether longer durations would eventually reveal group differences. Finally, no dietary recording/analysis was performed, leaving confounding issues such as calorie intake [28] unaddressed.

Thus, of the two known studies specific to strength athletes, neither was able to detect renal damage related to protein intake. Nonetheless, more evidence will be needed to address the concerns still present in educational materials. The totality of the literature appears to be a sum of 48 relatively-high-protein consuming strength athletes, compared to subjects unlike themselves, after fairly short (or unknown) periods of intake. Because strength athletes in particular routinely seek dietary protein [7] and they differ in training stresses, muscle mass, and dietary practices, there is a need for longer term study exclusively on this population. Lastly, the existing studies were done in European cultures with subjects who may eat differently than American students and strength athletes (to whom much protein dissuasion is targeted). Cultural differences in protein sources (e.g. amino acid profile, accompanying nutrients) could affect renal results when studying free-living persons [8]. Such potential cultural-dietary differences should be investigated among resistance trainers. We cannot assume that, when it comes to diet, "people are people". More homogeneous comparisons, still tighter experimental controls and longer study durations will help reduce the protein controversy currently in existence. Although not ideal from a cause: effect perspective, observational studies of long-time strength athletes would improve our understanding of the dietary protein-renal issue.

## Protein intake and bone health of athletes

Regarding calcium excretion, protein type (i.e. amino acid profile) again may matter. Recent evidence from Dawson-Hughes and colleagues (2007) suggests that specific amino acids are responsible for calciuric effects by binding to the calcium sensing receptor (CaR) [5]. After two weeks on a low-protein diet, healthy subjects received either a five-fold increase in aromatic amino acids (histidine, phenylalanine, tryptophan, tyrosine) or branched chain amino acids (leucine, isoleucine, valine) for two weeks. Both 24-hour and 4-hour calcium excretion after an amino acid load increased more in subjects receiving the aromatic amino acids. Interestingly, bone turnover markers did not change and the authors concluded that increased calcium absorption, rather than bone resorption (catabolism) was the likely cause. This conclusion differs greatly from the popular view that protein weakens bone [2,6].

Beyond amino acid profiles, other dietary constituents have an effect on bone metabolism. Clearly, calcium, vitamin D and phosphorous intakes are important, as often pointed out when comparing fracture risk among various populations [28,30]. Also, the effects of fruit and vegetable intake (e.g. potassium and alkalizing anions) are suspected to be beneficial to bone metabolism, outweighing the relatively minor ability of protein to acidify urine [30]. Conversely, saturated fat appears detrimental to bone density [31]. Purposefully sought ample protein intake, as part of a planned athletic diet, often involves food choices (e.g. low-fat dairy products and potentially vegetables) that provide the former nutrients but may or may not involve the latter nutrients (i.e. from fatty meats, egg yolks, full fat dairy, etc.). Dietary relationships are discussed in the final section of this review.

Specific to resistance-trained athletes, it is clear that the mechanical stimulus and/or blood flow changes induced by the exercise provides a strong stimulus for bone retention and anabolism [32]. Indeed, mechanisms are being increasingly clarified and exercise guidelines suggested [32,33]. Exercise appears even more important than diet regarding bone strength, a fact that emphasizes the strong bone-related differences exhibited by the resistance trained population. According to Specker and Vukovich, 2007: "...exercise would appear to be more important for optimizing bone strength because it has a direct effect (e.g. via loading) on bone mass and structural properties, whereas nutritional factors appear to have an indirect effect (e.g. via hormonal factors) on bone mass" [32].

It is not surprising that existing sports nutrition reviews do not include specific references to weight trained athletes when concluding that ample protein intakes are of little concern. Indeed, the authors of this review know of no research that has compared bone health (bone mineral content and density) in a group of resistance trainers who have or have not sought ample dietary protein over a multi-year period. This is important as years, not weeks, are required to assess done density change. As with renal evidence, well-controlled observational (cross sectional) studies in strength athletes, involving long-duration protein intakes could help. Again, the current and conspicuous absence of data is important because "education" provided to this population – which exhibits known improvements in bone strength – still often includes concerned or dissuasive language [2]. Researchers have reported and critiqued the common occurrence of bone health warnings in the media [6]. Why do the warnings persist?

## Protein's impact on other dietary parameters in athletes

The final category that will be addressed in the review is the impact of ample and purposefully sought protein intake on other dietary parameters. One critique that appears in educational materials such as some dietetic textbooks and personal trainer resource manuals is that higher protein diets are associated with higher total fat and saturated fat intakes and lower fiber consumption. (Table 1.) Do athletes differ here as well? A 1999 review by Hu and colleagues (2005) did reveal some, albeit weak, correlations (Pearson r = -0.11 to 0.24) between these variables among a large group of middle-aged, essentially healthy women [34]. Yet, is it appropriate to assume that athletes and resistance trainers in particular (who may seek additional protein for muscle building purposes) also follow these dietary trends? The United States diet is comprised of 42.2% meat, fish, poultry, 20.3% dairy, 4.2% egg, 9.8% vegetable, 4.1% legumes, nuts, seeds and 18% grains,[35] often including lower quality meats (e.g. fast food hamburgers). Would bodybuilders, for example, eat in this way? Or might they seek skinless chicken breasts instead of fatty hamburgers, skim milk and cottage cheese instead of 2% or whole dairy products, mostly egg whites instead of whole eggs, etc.? Again, the unfortunate state of the readily accessible literature is that despite concerned or dissuasive public education, there is a dearth of population-specific evidence.

## Summary

Existing health and safety education on dietary protein, including that geared toward athletes, is not entirely congruent with the relatively small amount of direct scientific evidence on the topic. There have been attempts to review the literature but often controversy, debate, misinformation, and a lack of needed evidence is observed [1,4,6,7]. The International Society of Sports Nutrition position statement, being the most recent sports nutrition-focused review on dietary protein safety, presented a balance of positive and negative literature on dietary protein but did not include safety data specific to athletes. Healthy sedentary persons differ from athletes in a number of ways. The omission of athletic data in such reviews is not surprising as few studies have been performed and none, to the knowledge of this review's authors, have documented long term (multi-year) effects of purposefully-sought dietary protein among athletes. This review has sought to describe the small amount of existing safety data specific to (resistance) athletes and point out where apparently none exist.

There are potential problems with the uncertain or potentially misguided language seen in the educational materials of several recent textbooks and of resources offered by sports governing bodies. (Negative verbal commentary surrounding the protein issues described herein is difficult to document and as such has been left from this review.) In any case, without evidence, one must wonder from where the dissuasive "education" stems. Various researchers have observed the disconnectedness between scientific evidence and public education regarding protein. The lack of population-specific data on athletes and the equivocal nature of existing data on non-athletes (e.g. elderly and even chronic kidney disease patients, beyond the scope of this review) bring into question why there is a "widely held belief that increased protein intake results in calcium wasting"[6] or why "Media releases often conclude that "too much protein stresses the kidney" [4]. Conversely, any conclusions that purposeful consumption of ample or surplus dietary protein are harmless or entirely without consequence are similarly under-substantiated, at least regarding the resistance trainer population. Note that the recent ISSN position paper quoted earlier in this review simply concludes that concerns are "unfounded" for healthy exercisers, not that a harmless situation exists. This is correctly cautious. Absence of evidence is not evidence of absence (regarding available data on protein's renal, bone or dietary consequences). As a population that routinely consumes higher amounts of protein,[7] strength athletes appear to be dismissing warning messages from educators but may instead be relying on questionable personal or anecdotal "evidence" once that educator credibility is lost. It would be truer to promulgate a message that the scientific and professional communities still lack specific information on the total safety profile of ample, purposefully sought protein among weightlifters. After decades of controversy we still simply do not explicitly know.

## Competing interests

The authors declare that they have no competing interests.

## Authors' contributions

LL was responsible for conceptualizing the review, directing the project, searching and reviewing scholarly materials, and drafting the majority of the manuscript. LD participated in searching and reviewing scholarly databases and textbooks as well as contributing to the methodology and assisting in coordination of the project. Both authors read and approved the final manuscript.
